# Robotic Radical Prostatectomy in Patients with Previous Prostate Surgery and Radiotherapy

**DOI:** 10.1155/2014/367675

**Published:** 2014-07-09

**Authors:** Ömer Acar, Tarık Esen

**Affiliations:** ^1^Department of Urology, VKF American Hospital, 34365 Istanbul, Turkey; ^2^School of Medicine, Koc University, 34450 Istanbul, Turkey

## Abstract

Herein, we will review the available literature about robot-assisted radical prostatectomy in patients who have undergone prostate surgery or radiotherapy. Current data about this topic consists of small case series with limited follow-up. Despite being technically demanding, robot-assisted radical prostatectomy (RARP) can be considered feasible in either setting. Prostate surgery or prostatic irradiation should not be considered as a contraindication for robot-assisted radical prostatectomy. Nevertheless, patient counseling about the possible complications and the need for reintervention is of extreme importance in this patient population. Early oncologic and functional results of RARP performed in case of radiorecurrent prostate cancer look promising. Regarding postprostate surgery RARP, some series have reported comparable results, while some have demonstrated more inferior outcomes than those of naive cases. In order to assess the exact functional and oncologic outcome of RARP in patients with previous prostate surgery and radiotherapy, studies enrolling higher number of patients and providing longer follow-up data are needed.

## 1. Introduction

Radical prostatectomy (RP) after previous treatments to the prostate such as transurethral resection of the prostate (TURP), photoselective vaporization (PVP), external-beam radiotherapy (EBRT), and brachytherapy (BT) is widely considered to be a more challenging task than in a prostate that has not been manipulated surgically or affected by radiotherapy. It is considered that previous prostatic resection or irradiation may hinder optimal results for radical prostatectomy in several ways. Most importantly, perioperative difficulties in defining correct surgical planes will increase the likelihood of significant complications and positive surgical margins. Additionally, functional outcomes related to erectile function and continence are at risk, due to the relative inability to identify and preserve neurovascular structures.

With increasing experience and the proven efficacy of robot-assisted radical prostatectomy (RARP), more complex cases such as those with previous TURP or radiotherapy (RT) are also being investigated using this technique. This review will focus on the perioperative, functional, and oncologic outcomes of robot-assisted radical prostatectomy, performed after prostate surgery or radiotherapy.

## 2. Robot-Assisted Radical Prostatectomy after Radiation Failure

### 2.1. Radiotherapy for Prostate Cancer

In localized prostate cancer (T1c-T2 N0 M0), three-dimensional (3D) conformal radiotherapy, with or without intensity modulated radiotherapy (IMRT), is recommended even for young patients who refuse surgical intervention. Irradiation offers the same long-term survival results as radical prostatectomy; moreover, EBRT provides a quality of life (QoL) at least as good as that provided by surgery [1]. Transperineal low-dose rate BT is another safe and effective technique that might be used to treat low-risk prostate cancer [2]. The eligibility criteria are stage cT1-T2a N0 M0, a Gleason score of 6 or lower assessed on a sufficient number of random biopsies, an initial PSA level of 10 ng/mL or lower, 50% or fewer of biopsy cores involved with cancer, a prostate volume of 50 mL or less, and a good IPSS. Recurrence-free survival after 5 and 10 years has been reported to range from 71% to 93% and from 65% to 85% [2].

According to the American Society for Therapeutic Radiology and Oncology (ASTRO) criteria, biochemical failure is defined as a PSA rise of 2 ng/mL above the posttreatment nadir, whether or not the patient received hormonal therapy in conjunction with radiation therapy [3].

Routine prostate biopsy should not be ordered for the evaluation of PSA-only recurrences, according to an ASTRO consensus recommendation [3]. On the other hand, prostate biopsy documenting local recurrence represents the main cornerstone in the decision-making process for salvage RP in patients with rising PSA levels following a nadir after radiation therapy. It is a general recommendation to wait about 18 months after radiation therapy or seed implantation [4]. Patients with rising PSA and viable cancer on biopsy 2 years after radiation therapy have true locally recurrent disease and might be candidates for salvage RP.

### 2.2. Therapeutic Options after Radiation Failure

In case of radiorecurrent PCa, treatment options include salvage radical prostatectomy (SRP), cryotherapy, high-intensity focused ultrasound (HIFU), and salvage BT [4, 5]. According to data from the Cancer of the Prostate Strategic Urological Research Endeavor (CaPSURE) database, 5277 men with PCa underwent RP (4342 men) or RT (935 men). In the latter group, 587 patients (63%) developed recurrent disease or received secondary treatment after a mean follow-up of 38 months [6]. Ninety-two percent of the patients in the CaPSURE database, who had radiorecurrent prostate cancer, received systemic ADT while only 2% of the patients underwent salvage RP [6]. Thus, more than 90% of the patients were placed on salvage hormonal therapy which is noncurative, indefinite, and associated with its own side effects and quality of life concerns such as hot flashes, sexual dysfunction, reduction in muscle mass, increased fracture risk, insulin resistance, and cardiovascular morbidity. The underutilization of salvage RP may be explained by its technically demanding nature and the fact that RT-induced cystitis, fibrosis, and tissue plane obliteration can increase the probability of complications with rectal injury, urinary incontinence, erectile dysfunction, and anastomotic contracture being reported in a substantial proportion of the cases [7–9]. However, in well-selected patients, the procedure may result in long-term disease-free survival.

### 2.3. General Considerations about Salvage Radical Prostatectomy

A candidate for salvage RP should fulfill the following requirements: surgically curable disease at initial radiation, no evidence of metastatic disease, a postradiation biopsy confirming PCa, and a life expectancy long enough (≥10 years) to benefit from intervention. Presurgical PSA (<10 ng/mL) and Gleason score of the postradiation biopsy (≤7) should also be considered when selecting a patient for salvage therapy [4, 10]. The first series of salvage RP and cystoprostatectomy following radiation failure was published in 1985 by Mador et al. [11]. The initial series reporting on salvage RP have included patients treated in the pre-PSA era without modern radiotherapeutic techniques, when local recurrences were usually detected at a late stage. Consequently, as high as 65% of the patients suffered from treatment-related morbidities. Up to 60% of patients who underwent salvage RP had to undergo anterior or total exenteration for locally extensive disease, associated with a high rate of local recurrences and a mean time of progression of only 1.3 years [4, 12]. Since then, salvage surgery has been used with increasing relative success.

Reports that have been published in the era of modern radiotherapy have described far more optimistic outcomes after salvage RP. In the recent literature review analyzing the currently available data on salvage RP, Chade et al. [10] reported that positive surgical margins varied from 43% to 70% in the earlier series and from 0% to 36% in the later studies (publication date before or after 2000). Pathologic organ-confined disease was reported in 22–53% and 25–81% of studies, respectively. At 10 years, biochemical recurrence-free probability ranged from 28% to 53%. Series from single centers reported probabilities of cancer-specific survival (CSS) from 70% to 79% at 10 years, while the multi-institutional study reported an 83% CSS at 10 years. Overall survival (OS) varied from 54% to 89% at 10 years [10].

Before SRP, PSA may be considered as the strongest prognostic factor predicting the oncologic outcome after salvage RP, because it was shown to significantly predict progression-free survival (PFS), CSS, and OS in most of the series [13–16]. Clinical stage before the commencement of RT [17], Gleason score prior to salvage RP, percentage of positive cores at biopsy, PSA doubling time, and low-dose brachytherapy were the other reported variables that have the potential to influence the oncologic outcome [18–20].

Regarding perioperative results and surgical complications, rectal injury ranged from 0% to 28% in patients and anastomotic stricture from 7% to 41%. Major complications (Clavien 3–5) occurred in 0–25% of patients, and estimated blood loss (EBL) varied between 119 mL and 1 L. Blood transfusion rates were found to be similar to the standard RP procedures [10].

Post-SRP erectile function sufficient for sexual intercourse ranged from 0% to 20% which led to the recommendation for oral medication or surgical treatment for ED in at least 80% of patients. Post-SRP urinary continence, defined by zero pads after SRP, ranged from 21% to 90% [10].

### 2.4. Salvage Laparoscopic Radical Prostatectomy

The feasibility of performing salvage prostatectomies has been extended into laparoscopy and more recently into robotic platforms [21, 22]. In 2003, Vallancien et al. were the first to report the outcome of laparoscopic SRP in seven cases with radiorecurrent PCa. Average operating time was 190 min (170–210 min). Two patients had positive surgical margins (PSM). At follow-up of 7, 12, 15, and 21 months, four patients were free of disease. No complications were reported. Five patients were continent and two patients who were potent before surgery became impotent [21]. Stolzenburg et al. investigated the feasibility of salvage endoscopic extraperitoneal RP for locally recurrent PCa in 9 patients. Regarding complications, only one patient with urinary retention was reported. Biochemical recurrence was recognized in only one patient 12 months postoperatively. Seven patients were completely continent [23]. Liatsikos et al. reported the outcome of another series of 12 patients who underwent salvage endoscopic extraperitoneal RP. Average operative time was 153 min, and mean blood loss was 238 mL. No intraoperative complications were reported. Biochemical recurrence was observed in one patient 12 months postoperatively. 10 patients were completely continent, but 3 preoperatively potent patients became impotent after the operation [24].

### 2.5. Salvage Robot-Assisted Radical Prostatectomy

Robot-assisted radical prostatectomy (RARP) is the most commonly performed surgical procedure for the treatment of patients with clinically localized prostate cancer in the United States. The robot offers several advantages, such as 3D vision, wristed instrumentation, motion scaling, image magnification, and tremor filtration. Hence, it may theoretically overcome the major limitations of salvage radical prostate surgery, such as the inability to identify correct surgical planes and neurovascular protection and therefore improve the oncologic and functional outcomes.

Avoidance of bladder neck contracture is very important after salvage RARP and, as a result of either the contracture or the treatment itself, these patients are at very high risk for incontinence. Watertight anastomosis with a running barbed suture may decrease the risk of anastomotic leakage and rate of bladder neck contractures.

Pneumoperitoneum may contribute to reduced blood loss compared to open salvage prostatectomy with EBL and transfusion requirements that do not appear to exceed that of primary (nonsalvage) robotic prostatectomy. However, all of these advantages may be offset by the lack of tactile feedback which is a major concern in this setting. Future improvements in haptic feedback technology are especially important in salvage surgeries where tissue planes are harder to discern.

In 2008, Jamal et al. reported a case of salvage robot-assisted radical prostatectomy (sRARP) [25]. The patient previously underwent 70 Gy of conformal EBRT and 3 months of antiandrogen treatment for a Gleason 6 tumor. He presented 2 years later due to hemospermia when he had a PSA of 0.7 ng/mL. Prostatic biopsy showed residual tumor and he underwent sRARP. After 3 months, the patient was continent, and his PSA was less than 0.03 ng/mL. After this initial report, several case-series have been published to date (Tables 1 and 2).

The first robotic series was reported by Kaouk et al. in which four patients underwent sRARPs with a mean operative time and EBL of 125 minutes and 117 mL, respectively. All patients had obturator lymph node dissection, and none showed any lymph node metastasis. The intraoperative courses were uneventful, but PSM occurred in two patients. At 1-month follow-up, 75% of the patients were dry on most days [22].

Subsequently, Boris et al. reported 11 patients who underwent sRARP with pelvic lymph node dissection. The mean operative time was 183 minutes, while the mean EBL was 113 mL. There were no intraoperative complications; however, an anastomotic leak developed in one patient necessitating extended catheterization, while an anastomotic stricture developed in another patient. Eight (72.7%) patients were continent (0-1 pad/d), of which six were pad free. Two patients had erections adequate for sexual intercourse. After a mean follow-up of 20.5 months, 3 patients had biochemical recurrence. They also supported the feasibility of sRARP [26].

Eandi et al. have reported their series of sRARPs consisting of 18 patients. Median operative time, EBL, and length of stay were, respectively, 2.6 h, 150 mL, and 2 days. Neither significant complications (rectal or ureteral injuries) nor need for blood transfusion was reported. Anastomotic extravasation was present in six (33%) cases, of which an anastomotic stricture developed in three. Five (28%) patients had a PSM, of whom two had organ-confined disease. One patient had lymph node metastasis. Six (33%) patients were completely continent, while all patients had ED after surgery. At a median follow-up of 18 months, 12 (67%) patients were free of biochemical recurrence [27].

Strope et al. reported the results of six patients who underwent RARP after definitive RT. The mean preoperative PSA level and the mean operative time were 9.3 ng/mL and 356 minutes, respectively. No rectal injuries or other intraoperative complications were experienced. Mean estimated blood loss was 280 mL. None received blood transfusion. Mean duration of hospitalization was 2 days. At 1 year, 2.3 pads per day were being used in the four patients who had eligible data regarding functional status. In three patients with available data at 1 year, all reported erectile dysfunction. Margins were negative in all but one patient and none of the patients had lymph node involvement. All except for 2 patients remained free of biochemical recurrence at a mean follow-up of 15 months [28].

Recently, Chauhan et al. retrospectively identified 15 patients who underwent sRARP in three academic institutions over a 20-month period with biopsy-proven prostate cancer after definitive radiotherapy. The median operative time, the median estimated blood loss, and the median length of hospital stay were 140.5 min, 75 mL, and 1 day, respectively. There were no rectal injuries. Two patients had a PSM. Of the patients, 71.4% were continent. At a median follow-up of 4.6 months, four (28.6%) patients presented with biochemical recurrence after sRARP [29].

Kaffenberger et al. reported their series of sRARP consisting of 34 patients who had surgery after the failure of prior definitive ablative therapy. Median time from primary therapy to sRARP was 48.5 months with a median preoperative PSA of 3.86 ng/mL. There were 2 Clavien grade 2 and higher complications. Postoperatively, 39% of patients were continent (0-1 pad/day). After a median follow-up of 16 months, 18% of patients had biochemical failure. The PSM rate was 26%, of which 33% had biochemical failure after surgery. On univariable analysis, PSA doubling time and Gleason score at original diagnosis were found to be significantly associated with biochemical failure. They highlighted the advantages of sRARP as superior visualization of the posterior prostatic plane, modest blood loss, low complication rates, and short length of stay [30].

Zugor et al. have reported the data of 13 patients who have undergone sRARP after failed external-beam radiotherapy (*n* = 7) or brachytherapy (*n* = 6). Neurovascular bundle preservation was performed in 3 patients (23.1%). Interestingly, they did not report any major intraoperative or postoperative complication. At 12 months, 7 patients were continent (53.8%), and 3 patients (23.1%) were potent. During the follow-up period, only 3 patients (23.1%) exhibited BCR [31].

The largest sRARP series has recently been published by Yuh et al. They reported the outcome of 51 consecutive patients who underwent sRARP after previous failed local therapy. Median age at surgery was 68 years and a median of 68 months has elapsed since primary treatment. Median operative time and estimated blood loss amount were 179 minutes and 175 mL, respectively. Half of the patients had extracapsular disease on eventual histopathologic assessment and the positive surgical margin rate was 31%. After a median follow-up of 36 months, their estimated 3-year biochemical recurrence or progression-free survival was 57%. Noteworthy, they reported significant complications (47%), incontinence, and erectile dysfunction rates. However, potency was maintained in 23% of preoperatively potent men and spontaneous return of urinary continence was achieved in 23 patients (45%) with a median time to continence of 6 months. Nevertheless, they emphasized the importance of proper patient counseling and selection before sRARP [32].

### 2.6. Summary

Salvage RP is a surgically challenging but effective secondary local treatment of PCa that recurred after radiotherapy with curative intent. Recommended criteria and identified predictive factors may help to select patients who are most suitable for salvage RP with long-term cure and good functional outcome. Extensive counseling regarding possible side effects of the procedure are mandatory.

In sRARP for radiorecurrent prostate cancer, tissue planes are often obscured and there is adherence of adjacent structures such as the rectum, pubic bone, and bladder. Healing and recovery are also delayed following energy destruction of tissue. Despite the fact that complications were not reported in a standardized categorical manner, the majority of them were of high importance as they required procedural intervention for bladder neck contracture, artificial urinary sphincter, or penile prosthesis.

Available data is consisting of small case series with limited follow-up. Larger series with a longer follow-up are necessary to make definitive conclusions about the oncologic and functional outcomes.

## 3. Robot-Assisted Radical Prostatectomy after Prostate Surgery

### 3.1. General Considerations

It is not uncommon for men to be diagnosed of cancer on TURP chips or to develop prostate cancer later after having undergone TURP for benign prostatic obstruction. Such men may have different outcomes on radical prostatectomy compared to those who have no such previous intervention. The relative paucity of the number of patients who underwent a previous TURP makes any comparative analysis difficult and also is a reflection of the few available studies on this subject in the literature (Tables 3 and 4).

There are several important factors which may affect the outcome of post-TURP RARP. The vesicourethral anastomosis may be technically difficult as a result of the rigidity of the bladder neck and the loss of elasticity of the urethra. Previous resection of the bladder neck could also distort the position of the ureteral orifices and thus increase the risk of ureteral injury. Increased periprostatic adhesions may increase the amount of blood lost and the risk of rectal injury during RARP.

The need for bladder neck reconstruction is increased because the bladder neck is wide, which may impact anastomotic urinary leakage and continence outcomes. TURP renders the internal sphincter mechanism deficient and also places the external sphincter at risk if the proper technique is not used which may increase the cumulative risk of postprocedural incontinence. Neurovascular bundle identification may be difficult as a result of periprostatic adhesions and therefore the risk of erectile dysfunction may be increased. Urethral manipulation and catheterization during TURP may result in anterior urethral stricture formation, a fact which may lead to infravesical obstruction and cause lower urinary tract symptoms after RARP.

Moreover, lymph nodes may be enlarged secondary to reactional changes post-TURP and may be difficult to interpret on preoperative imaging. There may be an increased risk of metastatic spread of disease because of the opening of lymphovascular channels during TURP along with continuous fluid irrigation.

### 3.2. Open and Laparoscopic Series

Most studies comparing the oncologic and functional outcomes in patients with and without previous TURP involve perineal, retropubic, laparoscopic, and endoscopic extraperitoneal RP series, data that ignore the advantages of the robotic technique. Colombo et al. evaluated the outcome of retropubic RP after TURP. They found a mean operative time from 135 to 105 minutes in the TURP group versus from 125 to 85 minutes in the control group. Complications were encountered in up to 50% of the patients. They reported that 74% and 86% of patients were dry at 6 and 12 months, respectively, and that 28% of patients were potent at 6 months. They considered RP to be feasible in patients after TURP [33].

Jaffe et al. compared the operative and postoperative outcomes of 119 patients who underwent laparoscopic RP after TURP to that of a randomized matched control group. Mean estimated blood loss, transfusion rate, and reoperation rates were statistically similar between the 2 groups. Postoperative length of stay (6.5 versus 5.2 days) and operative time (179 versus 171 minutes) were statistically higher in the TURP group. Positive surgical margin rate was significantly higher in the TURP group compared to the non-TURP group (21.8% versus 12.6%). Complications were observed in 64 patients in the TURP group compared to 34 in the non-TURP group (*p* < 0.01). Five (4.2%) and 2 (1.7%) men in the TURP and non-TURP groups, respectively, underwent reoperation during the postoperative period (*P* = 0.13) [34].

In a study by Teber et al., the authors evaluated the effect of previous TURP on surgical, functional, and oncologic outcomes after laparoscopic RP in comparison with a control group. The positive surgical margin rates were 14.5% and 16.3%, and the long-term continence rates were similar; however, previous TURP was associated with a lower continence rate (49.1%) at 3 months compared with 61.8% for the control group. There was no significant difference in the potency rates between the two groups at 12 months [35].

### 3.3. Robotic Series

Martin et al. were the first to investigate the effect of previous prostatic surgery on the outcome of robot-assisted RP. They evaluated the data of 24 patients who underwent RARP with a history of at least one previous treatment to the prostate including TURP, transurethral incision of the prostate (TUIP), transurethral microwave thermotherapy (TUMT), transurethral needle ablation (TUNA), photoselective vaporization of the prostate (PVP), simple prostatectomy, and open bladder neck reconstruction (group 1) and compared that of 486 patients with no treatment prior to RARP (group 2). Mean estimated blood loss, length of hospital stay, and operative duration were not statistically significantly different. However, the catheter time was significantly longer in group 1 (12 versus 8 days, *P* = 0.028). Complication rates were comparable (8.3% versus 6.8%). There were no bowel injuries in either group. The combined, overall PSM rate was 20.8% and 22.6% in groups 1 and 2, respectively (*P* = 1.0). Overall, there was no statistically significant difference in perioperative outcomes between the groups [36].

In the subsequent study conducted by Gupta et al., 158 cases of RARP for clinically localized prostate cancer, including 26 cases that had undergone previous TURP and 132 cases without TURP were analyzed in terms of surgical, oncological, and functional outcomes. The TURP cases were further subclassified into those who were found to have cancer after the histopathologic examination of TURP chips and those who had benign histopathology on TURP chips but developed cancer on subsequent follow-up. Post-TURP patients were found to have significantly greater blood loss (494 versus 324 mL) and a need for bladder neck reconstruction (26.7% versus 9.7%) compared to the non-TURP group. Conversion rates were also higher in TURP group (7.7%; 2/26) compared to non-TURP group (3%; 4/132). Importantly, all conversions in TURP were noted in those who had cT1b disease. Surgical time (189 versus 166 min), margin positivity rate, and biochemical recurrence rate were also higher in the TURP group. Regarding functional outcomes, incontinence rates in TURP group were higher both at 6 (14% versus 11.8%) and 12 (25% versus 8%) months follow-up than that of non-TURP group [37].

Zugor et al. compared the perioperative, oncologic, and functional outcomes of the patients who had undergone TURP before RARP (*n* = 80) and those who underwent RARP without a history of TURP (*n* = 80). Groups had equivalent clinicopathologic characteristics in this matched-pair analysis. Regarding preoperative characteristics, the only statistically significant difference was between potency rates (67.5% in TURP group versus 83.75% in non-TURP group, *P* = 0.056). The need for bladder neck reconstruction and operative time was significantly higher in the TURP group compared to the non-TURP group (58.7% versus 2.5%, *P* = 0.073 and 189 min versus 149 min, *P* = 0.069, respectively). The PSM rate did not exhibit statistical significance between two groups (6.25% versus 5%). Continence and potency rates in 12 months were similar (87.5%/91.25% and 70.3%/86.5%, respectively) [38].

Martinschek et al. compared the surgical outcome of robot-assisted radical prostatectomy and radical retropubic prostatectomy (RRP) in patients who underwent TURP. Mean operative time was 217 minutes for RARP and 174 minutes for RRP (*P* < 0.05). The overall positive surgical margin rate was 15.8% in both groups. Mean estimated blood loss was 33 mL in RARP patients and 1103 mL in RRP patients (*P* < 0.001). The RARP and RRP groups also differed in terms of hospital stay (8.58 versus 11.74 days; *P* = 0.0037), duration of catheterization (7.95 versus 11.78 days; *P* = 0.0016), postoperative complications according to the Clavien classification system (6 versus 15 patients; *P* = 0.0027), and transfusion rate (0% versus 10.5%; *P* < 0.001). They concluded that robot-assisted radical prostatectomy may be more feasible while dealing with prostate cancer patient who had undergone TURP [39].

Several newer modifications of TURP (e.g., transurethral vaporization resection of the prostate, bipolar TURP) and several types of laser prostatectomy (e.g., photoselective vaporization, holmium laser enucleation of the prostate, etc.) are available with each having its advantages and disadvantages. It may be possible that that these different technologies produce different degrees of prostatic, periprostatic, and bladder neck changes and may, therefore, have different degrees of impact on the outcomes of RARP. A recent study has reported on the feasibility of RARP after holmium laser enucleation of the prostate [40]. Apart from the technology used to resect the prostate, there are several other factors that may influence the final outcome. These include the size of the prostate gland, resection time, depth and completeness of resected prostate, presence of prostatitis, infection, surgeon's technique, and an individual's immune response. These factors directly affect the degree of inflammatory response in and around the prostate after TURP and may play important roles with respect to individual patient outcome.

### 3.4. Summary

The existing literature deals mainly with the post-TURP open and laparoscopic radical prostatectomies. Data is relatively scarce about the outcomes of RARP performed after prostate surgery. Some of the papers suggest that relatively poorer outcomes are achieved in men with previous pelvic or prostatic surgery. There are also suggestions that although surgery may be technically more difficult, overall morbidity, complications, and long-term functional or oncological outcomes are not different between these groups.

In conclusion, RARP is feasible after TURP, with good overall results being achieved. However, previous prostate surgery can cause difficulties for the surgical team during radical prostatectomy. Although previous TURP should not be considered as a contraindication for RARP, patients should be made aware of the above facts before undergoing RARP.

## Figures and Tables

**Table 1:** Perioperative outcomes in sRARP series.

Series	Patients, *n*	Type of radiation	Mean follow-up, mos	Mean EBL, mL	Mean OR time, min	Mean days on catheter, d	Mean hospital stay, d	Nerve sparing, *n*
Jamal et al. [25]	1	1 XRT	3	100	150	14	1	0
Kaouk et al. [22]	4	2 BT, 2 XRT + BT	5	117	125	15	2.7	0/4
Boris et al. [26]	11	6 BT, 4 XRT, 1 XRT + BT	20.5	113	183	10.4	1.4	NA
Eandi et al. [27]	18	8 BT, 8 XRT, 2 PBT	18	150	156	14	2	NA
Strope et al. [28]	6	4 XRT, 2 BT	>12	280	356	NA	2	1
Chauhan et al. [29]	15	5 XRT, 3 XRT + BT, 2 PBT, 5 BT	4.6	75	138	NA	1	2/15
Kaffen- berger et al. [30]	34	13 BT, 11 XRT, 6 BT + XRT, 4 HIFU	16.1	NA (88% had EBL ≤ 250 mL)	176	NA	NA (94% discharged on day 1)	NA
Yuh et al. [32]	51	22 BT, 18 XRT, 6 PBT, 3 Cryo, 1 HIFU, 1 XRT + BT	36	175	179	14	2	NA

EBL: estimated blood loss; OR: operative; XRT: external-beam radiation therapy; NA: not available; BT: brachytherapy; PBT: proton beam therapy; HIFU: high intensity focused ultrasound; and Cryo: cryotherapy.

**Table 2:** Oncologic and functional outcomes in sRARP series.

Series	Patients, *n*	Overall complications, *n*	Rectal injury, *n*	+LN, *n*	Continence, *n*	Potency, *n*	PSM, *n*	BCR during follow-up, *n*
Jamal et al. [25]	1	0	0	0	1/1	NA	0/1	0
Kaouk et al. [22]	4	1	0	0	3/4	NA	2/4	NA
Boris et al. [26]	11	3	0	2	8/11	2/11	3/11	3
Eandi et al. [27]	18	7	0	1	6/18	0/18	5/18	6
Strope et al. [28]	6	4	0	0	0/6	0/6	1/6	2
Chauhan et al. [29]	15	4	0	1	10/14	0/15	2/15	4
Kaffen-berger et al. [30]	34	13	1	0	12/33	5/33	9/34	6
Yuh et al. [32]	51	24	1	3	23/51	6/13	16/51	10

EBL: estimated blood loss; OR: operative; LN: lymph node; PSM: positive surgical margin; BCR: biochemical recurrence; and NA: not available.

**Table 3:** Perioperative outcomes in clinical series consisting of patients who underwent RARP after prostate surgery.

Series	Study population	Mean follow-up, mos	Mean EBL, mL	Mean OR time, min	Mean days on catheter, d	Mean hospital stay, d	Nerve sparing, *n*
Zugor et al. [38]	80 pts with TURP before RARP versus 80 pts with RARP	13.5	165 versus 144	189 versus 149	6.8 versus 5.2	NA	42 versus 54
Martin et al. [36]	24 pts with previous treatment to the prostate and RARP versus 486 with no previous treatment and RARP	NA	155 versus 137	200 versus 186	12 versus 8 (*P* = 0.028)	2.2 versus 1.5	14 versus 403
Gupta et al. [37]	18 pts with TURP before RARP versus 8 pts with pT1b CaP and RARP versus 132 pts with RARP	15.3	4941 versus 358.3 versus 324.2 (*P* = 0.03)	189.1 versus 162.5 versus 166	15.5 versus 14.2 versus 13.3	4.4 versus 4.1 versus 3.2	NA
Martin- schek et al. [39]	19 pts with previous TURP and RARP versus 19 pts with previous TURP and RRP	NA	333 versus 1103 (*P* < 0.001)	217 versus 174 (*P* = 0.02)	7.9 versus 11.7 (*P* = 0.001)	8.5 versus 11.7 (*P* = 0.003)	14 versus 13

TURP: transurethral resection of the prostate; RARP: robot-assisted radical prostatectomy; CaP: prostate cancer; EBL: estimated blood loss; OR: operating; and NA: not available.

**Table 4:** Oncologic and functional outcomes in clinical series consisting of patients who underwent RARP after prostate surgery.

Series	Study population	Transfusion, *n*	Rectal injury, *n*	+LN, *n*	Continence, *n*	Potency, *n*	PSM, *n*	BCR during f/u, *n*
Zugor et al. [38]	80 pts with TURP before RARP versus 80 pts with RARP	1 versus 0	1 versus 0	2 versus 0	70 versus 73	38 versus 58	6 versus 4	NA
Martin et al. [36]	24 pts with previous treatment to the prostate and RARP versus 486 with no previous treatment and RARP	0 versus 0	0 versus 0	NA	NA	NA	5 versus 110	NA
Gupta et al. [37]	18 pts with TURP before RARP versus 8 pts with pT1b CaP and RARP versus 132 pts with RARP	2 versus 1 versus 9	0 versus 0 versus 0	0 versus 0 versus 3	6/8 versus 5/6 versus 78/85	1/5 versus 1/3 versus 20/61	4 versus 1 versus 17	3 versus 1 versus 9 (*P* = 0.04)
Martinschek et al. [39]	19 pts with previous TURP and RARP versus 19 pts with previous TURP and RRP	0 versus 2 (*P* < 0.001)	0 versus 0	NA	NA	NA	3 versus 3	NA

TURP: transurethral resection of the prostate; RARP: robot-assisted radical prostatectomy; CaP: prostate cancer; LN: lymph node; PSM: positive surgical margin; BCR: biochemical recurrence; and NA: not available.
